# The Sheppard Hospital for the Insane

**Published:** 1891-10

**Authors:** 


### THE SHEPPARD HOSPITAL FOR THE INSANE.

THIS beneficent charity, erected by the liberality of one man, a public-spirited citizen of Baltimore, whose name it bears, is now nearly ready for the reception of patients. Dr. Edward N. Brush, a former resident of Buffalo, and for several years editor of this journal, has been chosen Superintendent. Last May a reception to the medical superintendents of insane hospitals in this country, and their friends, was given by the Board of Trustees, and we extract the following account thereof from the *Baltimore American* of May 2, 1891:

A special train, made up of eight coaches, left North avenue Station, on the Maryland Central Railroad, at 1.45 P. M. yesterday, for the Sheppard Asylum. On board were members of the Association of Superintendents of American Institutions for the Insane, and prominent citizens of Baltimore, who had been invited by the trustees to visit the Sheppard Asylum. Upon the arrival of the visitors at the asylum, luncheon was served.

Afterwards the visitors were addressed by Mr. George A. Pope, President of the Board of Trustees, who said the trustees had availed of the occasion of the meeting at Washington, D. C., of the annual convention of medical superintendents of the institutions for the insane of the United States, to invite its members to visit and inspect our work done at the Sheppard. Mr. Pope then gave a graphic account of the founding, plan and purpose of the institution. "With a few months' more work," he said, "on the water-fixtures, electric lights, mantels, interior furnishings, etc., the trustees expect to be ready to receive patients in the autumn of this year. They esteem the institution especially fortunate in having secured the services of Dr. Edward N. Brush as its medical superintendent. We present to you today merely the shell, the opportunity, of what may develop into a great curative establishment. You will see that much is yet to be done in furnishing and adornments inside, and in indispensable embellishments outside, such as walks, arbors, fountains, conservatories, flower and fruit gardens, lawns, pavilions for amusements, etc., and that our limited income will necessitate all this to be done gradually, and that to accomplish the approximate perfection we aim at, we shall require adequate compensation for the conveniences and facilities we can offer patients who can afford to pay for them, in order that we may be able to extend its advantages to some of slender means. It is by no means the intent to confine its resources for cures to the rich, but that the unfortunates who may be wealthy may help their poorer brethren."

### CONTRASTING THE PRESENT WITH THE PAST.

Dr. Edward N. Brush, of Philadelphia, the medical superintendent-elect of the Sheppard Asylum, then spoke, saying among other things: "It may not be generally known, but the sum left by Moses Sheppard, and the institution and grounds which have been provided therefrom, represent a larger amount than has ever been contributed by any individual in this country for the care of the insane. The State of Maryland has, therefore, the proud distinction of possessing the only institution in the United States for the insane founded by the charity of one individual. In Europe a few such exist, but I have learned of but two or three. One, the Crichton Royal Asylum, Dumfries, erected by Mrs. Crichton to the memory of her husband. The foundation fund, £120,000, represents almost exactly the sum left by Moses Sheppard. It was not until a little more than ten years before Moses Sheppard took the first steps toward founding this institution that chains and shackles were removed from the insane of England. I dare say there are some here who can recall the asylums of fifty years ago. Let him contrast them with these wards, even in their present state, and then imagine carpets, fixtures, books, furniture, flowers, and singing birds, and he will exclaim: "Truly, the gospel of the care of the insane today is that of sweetness and light."

I can say nothing which will better show what an institution of this kind should be than to quote from Conolly's concluding words in his "Construction and Government of Asylums." He says:

"I hope my concluding words will be believed when I say that if the whole of the system which I have endeavored imperfectly to sketch be steadily persevered in,—no anger, no severity, no revenge, no deception, no disregard ever shown to the insane,—calmness will come, hope will revive, satisfaction will prevail. Some unmanageable tempers, some violent or sullen patients there must always be, but much of the violence, much of the ill-humor, almost all the disposition to meditate mischievous or fatal revenge or self-destruction, will disappear. Some of the worst habits that beset the poor lunatic will also be got the better of; cleanliness and decency will be maintained or restored; and despair itself will sometimes be found to give place to cheerfulness or secure tranquility. Constant intercourse and constant kindness can alone obtain their entire confidence; and this confidence is the very keystone of all successful management."

Since that was written, medicine has taken enormous strides, but nothing today in the medical and general care of the insane remains which is not covered and included by these few lines. Such be our guiding principle. When these doors, a few weeks hence, are opened for the reception of patients, will begin the real work of the asylum. To relieve, to restore, to protect, to tenderly care for the unfortunates who are brought to our doors, will then be our duty and privilege. Let us hope to establish here at once an hospital and an asylum, for I believe the functions of an institution of this character are two-fold—to restore to his home and friends, if possible, the victim of insanity; if not, to afford him an asylum and refuge, a protection for himself and the world.

The other speakers were: Dr. Daniel Clarke, of Toronto; Dr. H. P. Stearns, of Hartford, Conn., and Dr. J. B. Chapin, of Philadelphia.

